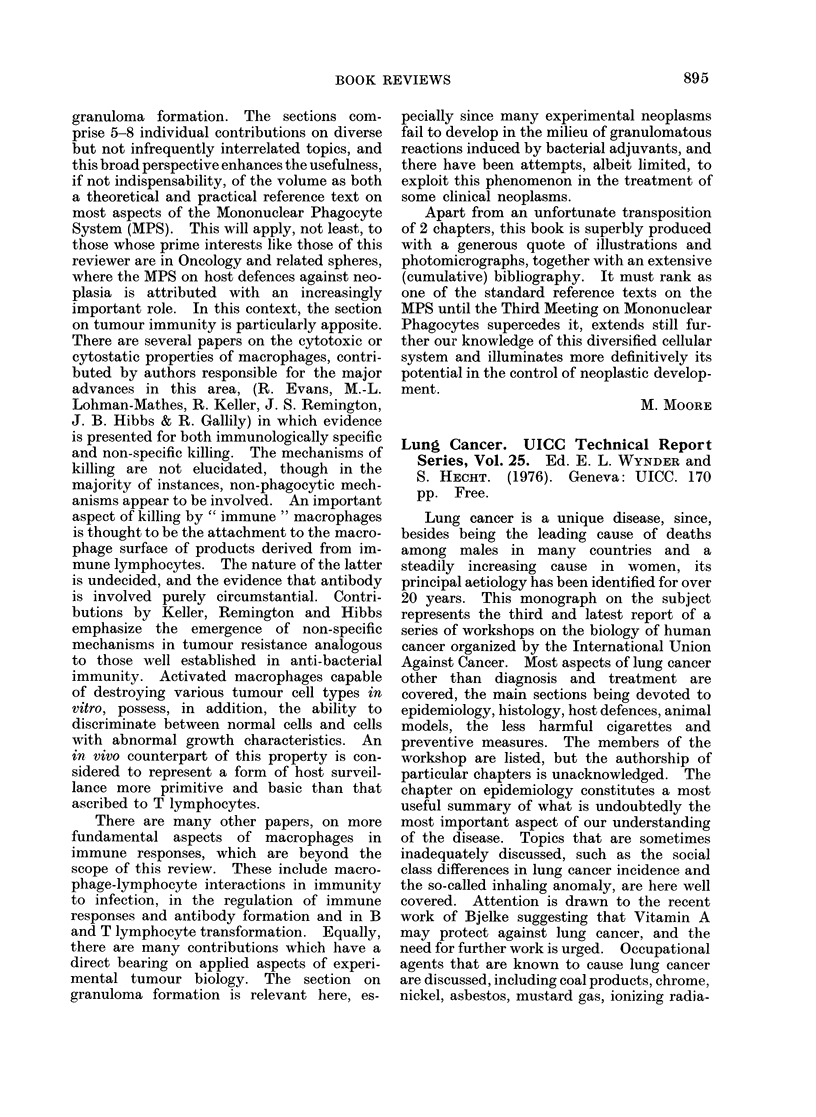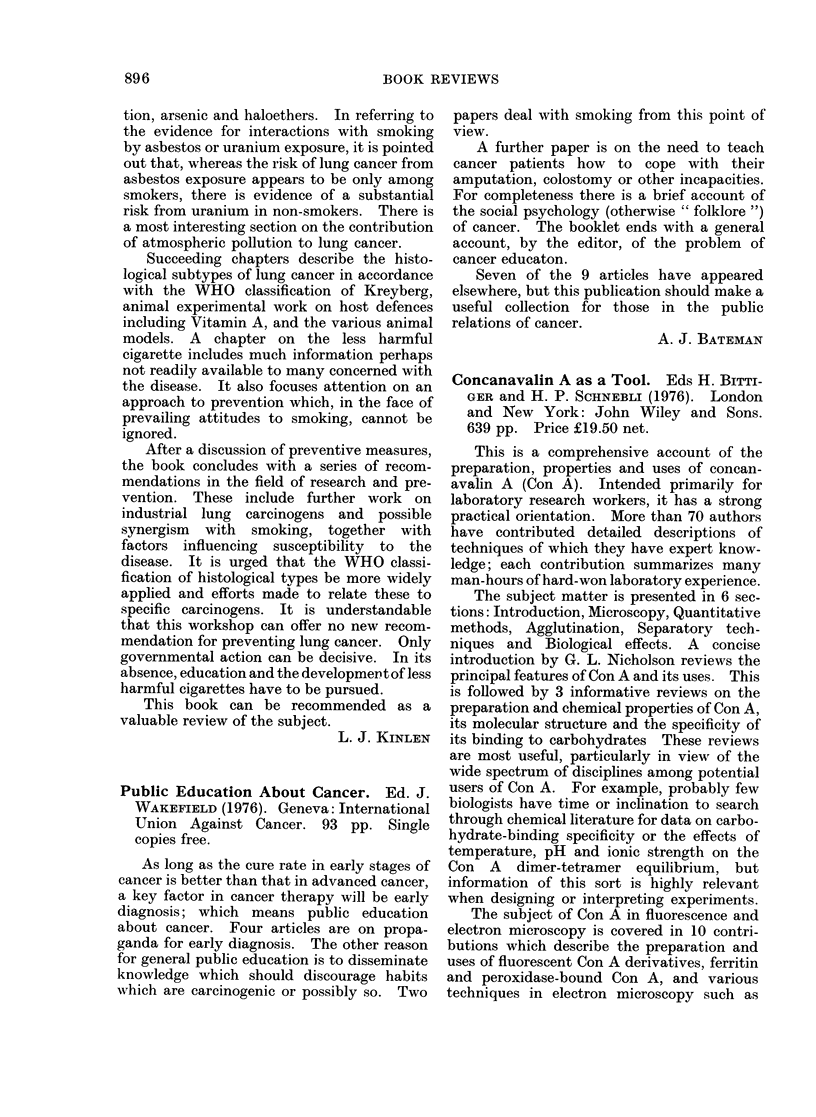# Lung Cancer. UICC Technical Report Series, Vol. 25

**Published:** 1977-06

**Authors:** L. J. Kinlen


**Lung Cancer. UICC Technical Report Series, Vol. 25.** Ed. E. L. WYNDER and S. HECHT. (1976). Geneva: UICC. 170 pp. Free.

Lung cancer is a unique disease, since, besides being the leading cause of deaths among males in many countries and a steadily increasing cause in women, its principal aetiology has been identified for over 20 years. This monograph on the subject represents the third and latest report of a series of workshops on the biology of human cancer organized by the International Union Against Cancer. Most aspects of lung cancer other than diagnosis and treatment are covered, the main sections being devoted to epidemiology, histology, host defences, animal models, the less harmful cigarettes and preventive measures. The members of the workshop are listed, but the authorship of particular chapters is unacknowledged. The chapter on epidemiology constitutes a most useful summary of what is undoubtedly the most important aspect of our understanding of the disease. Topics that are sometimes inadequately discussed, such as the social class differences in lung cancer incidence and the so-called inhaling anomaly, are here well covered. Attention is drawn to the recent work of Bjelke suggesting that Vitamin A may protect against lung cancer, and the need for further work is urged. Occupational agents that are known to cause lung cancer are discussed, including coal products, chrome, nickel, asbestos, mustard gas, ionizing radiation, arsenic and haloethers. In referring to the evidence for interactions with smoking by asbestos or uranium exposure, it is pointed out that, whereas the risk of lung cancer from asbestos exposure appears to be only among smokers, there is evidence of a substantial risk from uranium in non-smokers. There is a most interesting section on the contribution of atmospheric pollution to lung cancer.

Succeeding chapters describe the histological subtypes of lung cancer in accordance with the WHO classification of Kreyberg, animal experimental work on host defences including Vitamin A, and the various animal models. A chapter on the less harmful cigarette includes much information perhaps not readily available to many concerned with the disease. It also focuses attention on an approach to prevention which, in the face of prevailing attitudes to smoking, cannot be ignored.

After a discussion of preventive measures, the book concludes with a series of recommendations in the field of research and prevention. These include further work on industrial lung carcinogens and possible synergism with smoking, together with factors influencing susceptibility to the disease. It is urged that the WHO classification of histological types be more widely applied and efforts made to relate these to specific carcinogens. It is understandable that this workshop can offer no new recommendation for preventing lung cancer. Only governmental action can be decisive. In its absence, education and the development of less harmful cigarettes have to be pursued.

This book can be recommended as a valuable review of the subject.

L. J. Kinlen